# An Unusual Cause of Breast Pain

**DOI:** 10.5334/jbr-btr.895

**Published:** 2016-01-29

**Authors:** Marie-Axelle Van Caulaert, Isabelle Leconte, Latifa Fellah

**Affiliations:** 1 Cliniques Universitaires, St Luc, BE

**Keywords:** breast, ultrasound, pain, Mondor

We report the case of a 30-year-old woman, two pregnancies (first delivery at the age of 27), menarche at the age of 13 who presented to the emergency department for the recent occurrence of a right breast pain. There was a familial history of early onset of breast cancer (one maternal cousin at the age of 36). She had no personal history of breast pathology.

Clinical examination revealed a 5 mm-wide tubular induration of the upper lateral quadrant of the right breast (Fig. 1). The area was very sensitive to palpation, although it did not look inflamed. No homolateral adenopathy was found, neither was another lesion.

**Figure 1:**
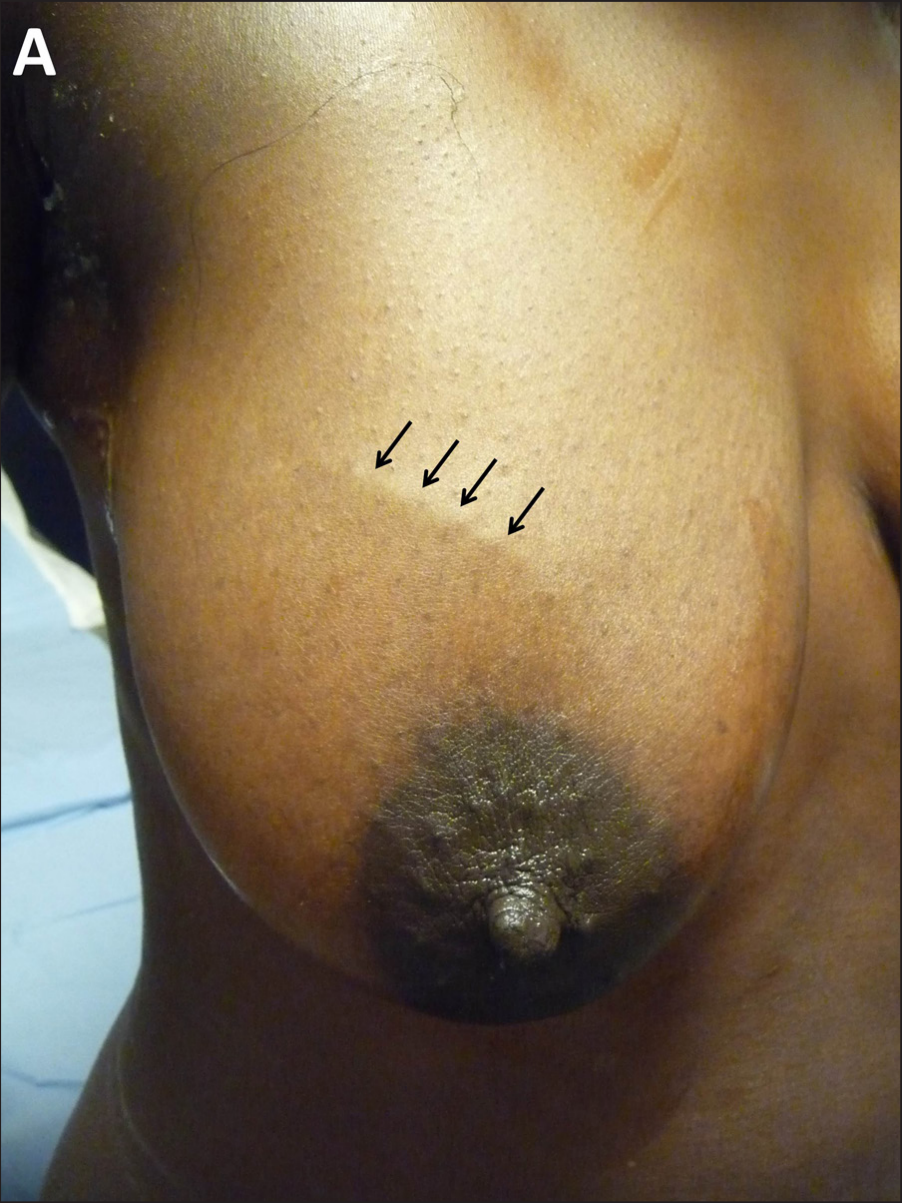
Tubular induration of the upper lateral quadrant of the right breast.

Breast ultrasound was performed and showed a hypoechogenic subcutaneous and uncompressible linear structure (Fig. 2). There was no visible flow with color Doppler ultrasound inside the lesion (Fig. 3). Altogether, we came to the diagnosis of a breast superficial vein thrombosis, also called Mondor’s disease.

**Figure 2:**
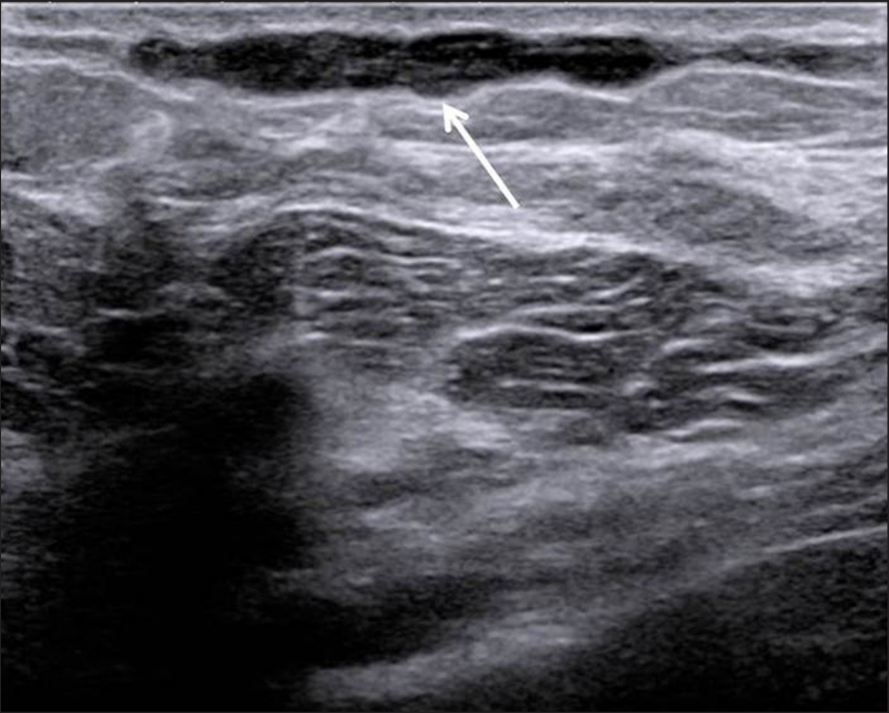
2D ultrasound: hypoechogenic subcutaneous and uncompressible linear structure.

**Figure 3:**
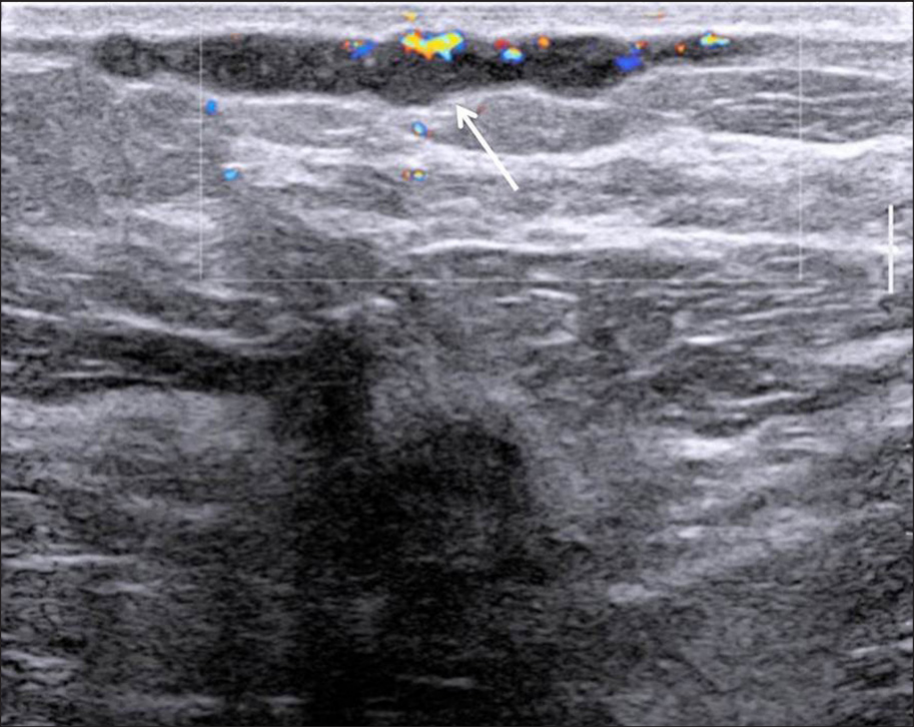
2D ultrasound with color Doppler: no vascularization inside the linear structure.

## Comment

Mondor’s disease is a rare benign condition described for the first time by Henri Mondor in 1939. It is a superficial thrombophlebitis of a subcutaneous vein of the anterior wall of thorax or abdomen. Risk factors include female gender, age between 30 and 50, recent thoracic trauma or surgery, intense physical activity, local infections, intravenous drug consumption and the presence of intravenous catheter [1]. Pregnancy and smoking have also been associated with Mondor’s disease. Except gender and age, our patient had no risk factor for Mondor’s disease.

Diagnosis is first based on clinical presentation and physical examination. It is usually confirmed by ultrasound which also excludes other conditions like tumor or canal dilation.

Mondor’s disease classically resolves spontaneously within a period from one to six months.

There is no consensus regarding management: painkillers are widely used, but the role of non-steroids anti-inflamatory drugs and anti-coagulation therapy is elusive and varies depending on the importance and the localization of the thrombosis. Recent publications suggest that patients might beneficiate from a short-term (1–2 months) treatment with low dose of low-molecular weight heparin.

A full coagulation check-up is advised, as this condition has been linked to several coagulopathies like anti-cardiolipin syndrome, and oral contraception intake.

## Competing Interests

The authors declare that they have no competing interests.
